# Editorial: Omics applied to livestock genetics: volume II

**DOI:** 10.3389/fgene.2024.1477826

**Published:** 2024-08-23

**Authors:** Lucas Lima Verardo, Nuno Carolino, Marcela Ramos Duarte, Emily Alves Rodrigues Almeida, Gabriel Dallago, Ana Fabrícia Braga Magalhães

**Affiliations:** ^1^ Laboratory of Animal Breeding, Department of Animal Science, Universidade Federal dos Vales do Jequitinhonha e Mucuri, Diamantina, Brazil; ^2^ Instituto Nacional Investigação Agraria e Veterinaria (INIAV), Oeiras, Portugal; ^3^ Department of Animal Science, University of Manitoba, Winnipeg, MB, Canada

**Keywords:** data integration, epigenomics, farm animals, genomics, multiomics, proteomics, transcriptomics

After the first sequencing of a mammalian genome, several studies have been published with a variety of omics datasets aiming to unravel the biological aspects that influence the phenotypic expression of complex traits (e.g., Gallagher and Chen-Plotkin, 2018; Costa et al., 2013; Legrain et al., 2011; Portela and Esteller, 2010; Marcobal et al., 2013; Fonseca et al., 2018). These studies revolutionized genome translation into phenome in the last 2 decades, including the development of important tools for the livestock sector. Projects, initiatives, and databases provide knowledge of genetic variations for the main traits of livestock species that are economically, environmentally, and socially important. For instance, the AnimalQTLdb project (Hu et al., 2007) has curated genomic information of many quantitative trait loci (QTL) identified in cattle, pigs, chicken, sheep, and other species.

The large-scale datasets generated by livestock “omics” projects have been made publicly available to researchers aiming to generate knowledge and translation tools for improving animal production and sustainability. For instance, the Functional Annotation of Animal Genomes (FAANG) project has generated datasets to decipher the function of genome segments, and it has analyzed samples from approximately 15 species, including pigs, cattle, sheep, and salmon (Giuffra et al., 2019). Moreover, the “omics” approaches can be holistically evaluated (Vieira et al., 2024) and applied to improve animal breeding strategies based on biology-driven genomic predictions, in addition of contributing to a better understanding of the genomic background of phenotypic variability in livestock systems (Chakraborty et al., 2022).

**FIGURE 1:**
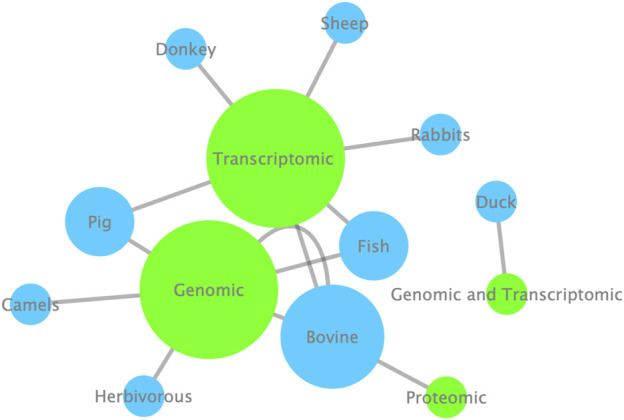
Enrichment analysis highlighting the most omic approaches (green nodes) used and the most livestock species (blue nodes) studied in the Research Topic. The size of the nodes corresponds to the Cytoscape network enrichment analysis; the bigger the nodes, the more used and/or studied the corresponding nodes.

The Research Topic titled “Omics Applied to Livestock Genetics II” presents a collection of the latest findings in livestock genetics based on omics approaches. Studies focusing on livestock animals, such as pigs, cattle, ducks, camels, rabbits, donkeys, and sheep, involving omics data revealed genetic information related to various relevant traits. In volume I of Omics Applied to Livestock Genetics, the two most used approaches were genomics and transcriptomics applied, with cattle and pigs being the main studied species. Now, at volume II, fish was also highlighted (Figure 1). The results presented in this Research Topic provide significant advancements toward understanding farm animal genetics

The genomic knowledge of many commercially important fish species is still incipient, as is the case with some catfish species. The study by Ye et al. provided the first high-quality chromosomal-level reference genome map for the *Hemibagrus macropterus*. Using homology-based, *de novo*, and RNA-seq methods, various genes were identified and functionally annotated through a functional database. The assembled genome will be beneficial for exploring genome evolution and sexual determination mechanisms as well as facilitate future comparative genomics and conservation studies in Siluriformes. In another study also using catfish as a model, Wang et al. analyzed different organs in two species of catfish. The study performed RNA sequencing (RNA-seq) experiments to establish transcriptomic resources for both species’ tissues (heart, liver, intestine, mucus, and muscle). A variable number of genes were identified as being expressed in each tissue, with a notable high expression of genes in the mucus. Gene ontology (GO) analysis revealed the functional specificity of differentially expressed genes in each tissue, with significant enrichment in metabolic pathways, immune activity, and stress responses. Tissue-specific genes such as *lrrc10*, *fabp2*, *myog*, *pth1a*, *hspa9*, *cyp21a2*, *agt*, and *ngtb* were identified. This study demonstrates that transcriptomics may be used to support further investigations into the molecular basis underlying environment-dependent heterosis and advance genetic breeding efforts of hybrid catfish

Studying camels, Abri et al. characterized the genetic diversity and selection signatures in camels from the Oman region. Using SNP (Single Nucleotide Polymorphism) genotyping data, genetic differentiation was observed due to evolutionary processes for adaptation between Muscat dromedaries and Al-Batinah and Al-Sharqiya populations. Candidate genes such as *SLC2A9*, *LEP*, and *PTPN22* were identified, which are involved in biological processes influencing the survival and reproduction of dromedaries in arid environments. For instance, *SLC2A9* is cited to be involved in glucose transport and may play a role in regulating energy metabolism. Genes associated with functional categories related to immune response, lipid metabolism, energy expenditure, optical and auditory functions, and long-term memory were also identified.

In this Research Topic, bovine was the most studied species. Rosyada et al. assessed the semen of native Indonesian bulls. Through proteomic investigations, they found that bull fertility is associated with many proteins involved in spermatogenesis. They identified 15 proteins linked to metabolic pathways and the tricarboxylic acid cycle, contributing to sperm energy production. Proteins related to thermal stress and predictors of thermotolerance, such as *HSPA9* and *HSPA2*, were identified as protective agents for sperm. Jang et al. studied Hanwoo cattle, a native breed from Korea known for high fertility but slow growth rates. This study collected samples from 22 different tissues of castrated males and utilized RNA-seq technology for gene expression profiling, integrating eQTL analysis to elucidate the genetic mechanisms influencing weight. By integrating the results from eQTL analyses and differentially expressed genes (DEGs), genomic regions that may regulate the expression of candidate genes, such as *TRIM31*, were identified. Reduced expression of *TRIM31* was associated with weight gain, which can be explained by cis-eQTL candidate genotypes and their effect on differential gene expression between lower and higher weight groups.

Also studying bovine specie, Wang et al. utilized Specific Locus Amplified Fragment (SLAF) sequencing to examine the genetic structure and diversity of Xinjiang Brown (XBG) cattle. Selection signature analysis revealed differentiated patterns between XBG and the ancestral breeds Swiss Brown (BS) and Kazakh (KZ). Besides, candidate genes enriched with GO terms related to disease resistance and the endocrine system were identified. This research enhances the understanding of genetic diversity in Xinjiang Brown cattle and provides valuable insights for future selection and genetic breeding practices. The fourth study using bovine as a model, Nawaz et al., sought to identify genomic regions under selection in Hanwoo and Angus cattle using advanced genetic analysis methods based on imputed whole-genome sequencing variants. The study employed allele frequency-based and haplotype-based methods, including runs of homozygosity and extended haplotype homozygosity, to detect selection signals within each breed and between the two breeds. In Angus cattle, 27 genomic regions housing 360 genes were identified. The identified genes are associated with growth, immunity, reproductive development, feed efficiency, and environmental adaptation, suggesting that selection processes in this breed focused on productivity and environmental adaptability. In contrast, Hanwoo cattle displayed 17 genomic regions containing 59 genes. Candidate genes indicated that selection in Hanwoo prioritized traits related to meat quality and sensory perception. This study enhances the understanding of the genetic architecture of selection in Angus and Hanwoo cattle, highlighting breed-specific adaptations and priorities.

Considering the studies focusing on pigs, Xiao et al. studied the Acetyl-Coenzyme A Acyltransferase-1 (*ACAA1*) gene, which is involved in fatty acid metabolism. The study assessed the mRNA expression levels of *ACAA1* in various tissues (heart, liver, spleen, lung, kidney) from 6-month-old Xiangsu pigs. The mRNA expression was also evaluated in the *Longissimus dorsi* muscle at different growth stages (newborn, 6 months, and 12 months) using RT-qPCR. Additionally, the relationship between single-nucleotide polymorphisms (SNPs) of *ACAA1* gene and growth traits in 6-month-old and 12-month-old Xiangsu pigs was investigated on 184 healthy Xiangsu pigs. The expression of *ACAA1* was detected in multiple tissues of 6-month-old Xiangsu pigs, with the highest expression observed in the liver. In the *L. dorsi*, expression decreased as the animals grew. Significant associations between SNPs and these growth traits suggest that the *ACAA1* gene is a potential marker for genetic selection in Xiangsu pigs. In another study, Wang et al. investigated the correlation between intramuscular fat content (IFC) and meat color (CIE) in pigs using an integrative approach that combines differential gene expression analysis, gene co-expression network analysis (WGCNA), functional enrichment analysis, and the identification of candidate and hub genes. The results revealed 485 and 394 DEGs and identified 47 and 53 candidate genes affecting IFC and CIE, respectively. Protein-protein interaction (PPI) network analysis of the candidate genes identified 5 hub genes affecting IFC and 13 hub genes affecting CIE value. Four crucial hub genes (*MYC*, *SOX9*, *CEBPB*, and *PPARGC1A*) were shared between these two traits. The authors propose that the *SOX9/CEBPB/PPARGC1A* axis may co-regulate lipid metabolism and the redox response of myoglobin, contributing to a better understanding of the molecular mechanism underlying the co-regulation of IFC and CIE value.

Using an integrative approach, the study by Li et al. on Jianshui yellow-brown ducks used resequencing and transcriptomic data and revealed several SNPs and InDels, with variants identified in genes associated with muscle development and fat metabolism. This study used phylogenetic trees, PCA, and admixture analysis to investigate the population genetic structure of Jianshui yellow-brown ducks by comparing their selection signals with those of ancestral mallard ducks and meat-type Pekin ducks. Selection signal analysis indicated significant selection pressure on genes related to meat traits (*ELOVL2*, *ELOVL3*, *GDF10*, *VSTM2A*, *PHOSPHO1*, and *IGF2BP*1) in Jianshui yellow-brown ducks and mallard ducks. While transcriptomic analysis suggested that *ELOVL3*, *PHOSPHO1* and *GDF10* are candidate genes influencing meat production and quality in Jianshui yellow-brown ducks.

Another species of significant economic interest presented in this Research Topic was the rabbit (*Oryctolagus cuniculus*), in which Jia et al. obtained samples from the *Longissimus dorsi* muscle of male and female rabbits and utilized Oxford Nanopore Technologies long-read sequencing technology to investigate the association between gene expression levels and growth traits through large-scale transcriptome-wide association studies (TWAS). This contributed to the improvement of rabbit genome annotation. However, the transcriptome-wide association studies did not identify statistically significant genes or transcripts associated with the growth traits examined, highlighting the need for other omics studies in this species aiming to a better understanding of growth traits’ genetic architecture.

Aiming to investigate the transcriptomic screening of lncRNAs and mRNA associated with skin development and collagen organization, Wang et al. obtained skin tissue samples from Dezhou donkeys at different stages, including the 8-month fetal stage, and at ages of 2 and 8 years. Through enrichment analyses and functional analyses, it was possible to identify specific lncRNAs and interactions between mRNA and lncRNA. Specific lncRNAs, including *ENSEAST00005041187*, *ENSEAST00005038497* and *MSTRG.17248.1*, which potentially regulate the *COL1A1* gene that is responsible for the type I collagen chain I, were identified through interaction networks. Collagen organization and skin development pathways were also observed, including protein digestion and absorption, metabolic pathways, phosphatidylinositol 3-kinase-protein kinase B signaling pathway (PI3K-Akt signaling pathway), extracellular matrix-receptor interaction (ECM-receptor interaction) and relaxin signaling and biological function processes. The *COL1A1*, *COL3A1* and *LOXL2* genes were involved in the regulation of these pathways.

Finally, Liu et al. reviewed Copy Number Variants (CNVs) in the genomes of herbivorous livestock species, including cattle, sheep, horses, and donkeys. They presented a brief elucidation of the fundamental concepts underlying CNVs, their mutational mechanisms, and the diverse array of detection methods that can be employed to identify these structural variations within genomes. The review highlighted the role of CNVs in shaping various phenotypic traits, including growth and reproductive traits, pigmentation, disease resistance, etc. In conclusion, the authors stated that CNVs represent a valuable and dynamic field of study poised to impact the genetic improvement of herbivorous livestock species, ultimately benefiting both human society and the global livestock industry.

As observed in the first volume, the main livestock species have been studied through omics approaches. However, multiomic analyses are still scarce, and the generation and sharing of multiomic datasets are crucial for further advancing research in this field. Functional genomic analyses and high-throughput phenotyping are relevant for providing a clearer picture of the genome-to-phenome paradigm in livestock systems. Moreover, the integration of omics technologies with phenomics into the breeding programs, which is still lacking in this Research Topic, may be helpful for increasing the rate of genetic progress in sustainable breeding programs.
